# Production of single chain Fab (scFab) fragments in *Bacillus megaterium*

**DOI:** 10.1186/1475-2859-6-38

**Published:** 2007-11-27

**Authors:** Eva Jordan, Laila Al-Halabi, Thomas Schirrmann, Michael Hust, Stefan Dübel

**Affiliations:** 1 Technische Universität Braunschweig, Institut für Biochemie und Biotechnologie, Abteilung Biotechnologie, Spielmannstr. 7, 38106 Braunschweig, Germany

## Abstract

**Background:**

The demand on antigen binding reagents in research, diagnostics and therapy raises questions for novel antibody formats as well as appropriate production systems. Recently, the novel single chain Fab (scFab) antibody format combining properties of single chain Fv (scFv) and Fab fragments was produced in the Gram-negative bacterium *Escherichia coli*. In this study we evaluated the Gram-positive bacterium *Bacillus megaterium *for the recombinant production of scFab and scFvs in comparison to *E. coli*.

**Results:**

The lysozyme specific D1.3 scFab was produced in *B. megaterium *and *E. coli*. The total yield of the scFab after purification obtained from the periplasmic fraction and culture supernatant of *E. coli *was slightly higher than that obtained from culture supernatant of *B. megaterium*. However, the yield of functional scFab determined by analyzing the antigen binding activity was equally in both production systems. Furthermore, a scFv fragment with specificity for the human C reactive protein was produced in *B. megaterium*. The total yield of the anti-CRP scFv produced in *B. megaterium *was slightly lower compared to *E. coli*, whereas the specific activity of the purified scFvs produced in *B. megaterium *was higher compared to *E. coli*.

**Conclusion:**

*B. megaterium *allows the secretory production of antibody fragments including the novel scFab antibody format. The yield and quality of functional antibody fragment is comparable to the periplasmic production in *E. coli*.

## Background

Recombinant antibody fragments are indispensible tools for research, diagnostics and therapy [1-3]. However, new areas of application and the increasing demand for recombinant antibodies requires new antibody formats as well as appropriate production systems. Particularly, if a large number of different antibody clones must be produced, for example for the functional genome analysis, bacteria are a very interesting antibody production system, because they offer simple medium demands, short production time and high capacity to produce recombinant proteins. However, the capability to produce functional full-size antibodies in bacteria is very limited. Therefore, smaller antibody fragments like the single chain Fv (scFv) and Fab are produced in bacteria. The scFv format is composed of the variable regions of the antibody heavy (VH) and the light chain (VL) linked by a 15–25 amino acid linker [4,5]. ScFvs have the tendency to form aggregates and are relatively unstable over longer periods of time [6]. Furthermore, some scFvs show a reduced affinity of up to one order of magnitude compared to the corresponding Fab fragments [7]. Only in rare cases scFvs with a higher affinity than the associated Fab have been found [8]. Fab fragments are composed of VH and VL fused to the following constant antibody domain of the heavy (CH1) and light chain (CL). Both chains are covalently linked by a disulphide bridge. Because they are double the molecular size, and require the production and connection of two different polypeptides with a disulphide bond, folding and assembly of Fab fragments in the periplasm of *E. coli *is less efficient than of scFvs [9]. A further disadvantage of Fab fragments is the tendency of the light chains to form homo-dimers, which are known as Bence Jones proteins [10]. Advantages of Fab fragments are their high stability while long term storage [11] and their compatibility with common detection antisera without the need for a re-engineering step [2]. Recently, the novel single chain Fab (scFab) antibody format was developed by Hust et al. [12] combining the advantages of scFv and Fab fragments. The scFab is composed of VH-CH1 and VL-CL connected by an 34 amino acid linker without the cysteins connecting both antibody chains (scFabΔC). The scFab was produced functionally in *E. coli *and *Pichia pastoris *[12].

Due to the lack of the outer membrane Gram positive bacteria have higher capacity for protein secretion into the medium in comparison to Gram negative *E. coli*. The Gram-positive bacteria *B. brevis *[13,14] and *B. subtilis *[15,16] have already been successfully used for the production of antibody fragments. In contrast to *B. subtilis*, *B. megaterium *does not produce alkaline proteases and has also a higher plasmid stability during growth [17], a prerequisite for stable gene expression in long term cultivations like in bioreactors. Recently, the first production of functional lysozyme specific scFv (D1.3) antibody fragments in *B. megaterium *was successfully demonstrated [18]. In this study, *B. megaterium *was used for the production of the novel scFab format and another scFv fragment and compared to *E. coli *production of the same antibody fragments.

## Results

### Construction of the vectors pEJBmD1.3scFab and pEJBmLA13-IIE3scFv

**Figure 1:**
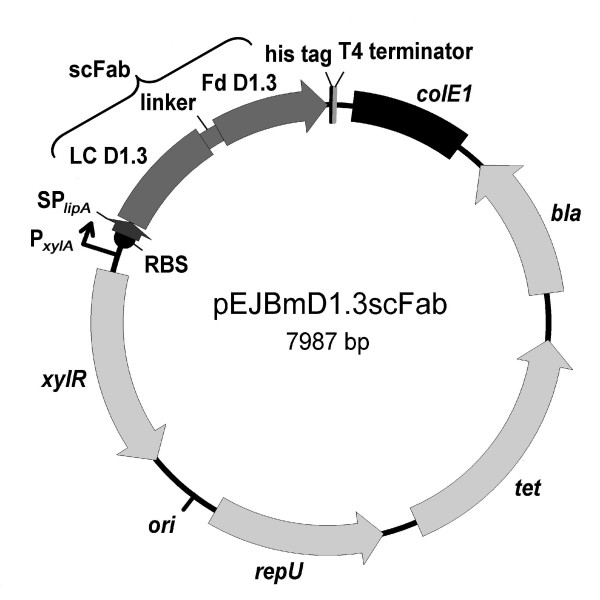
Plasmid map of pEJBmD1.3scFab. Abbreviations: *bla*: β-lactamase gene for ampicillin resistance; *colE1*: *E. coli *origin of plasmid replication; His-tag: synthetic 6xhistidine tag; ori: *B. megaterium *origin of plasmid replication; P_*xylA*_: xylose inducible promoter; RBS: ribosome binding site; repU: a gene for plasmid replication in *B. megaterium*; scFab: single chain fragment antigen binding; SP_lipA_: signal peptide sequence of *B. megaterium *extracellular esterase LipA; terminator: sequence terminating transcription; tet: tetracyclin resistence gene; LC: light chain; Fd: VH and CH1 of the heavy chain; *xylR*: xylose repressor

The anti-lysozyme scFab D1.3 (scFabΔC) DNA was amplified using polymerase chain reaction (PCR) from the vector pHAL1-D1.3-scFabΔC [12] using gene specific oligonucteotide primers and cloned between the restriction sites *Nhe*I and *Not*I of pEJBmopSplipA [18] resulting in the vector pEJBmD1.3scFab (Fig. 1). In the same way, the anti human C-reactive protein (CRP) scFv LA13-IIE3 obtained by phage display (Al-Halabi et al., unpublished) was amplified using gene specific oligonucleotide primer and cloned into pEJBmopSplipA [18].

### Production and analysis of D1.3 scFab in *B. megaterium *and *E. coli*

**Figure 2:**
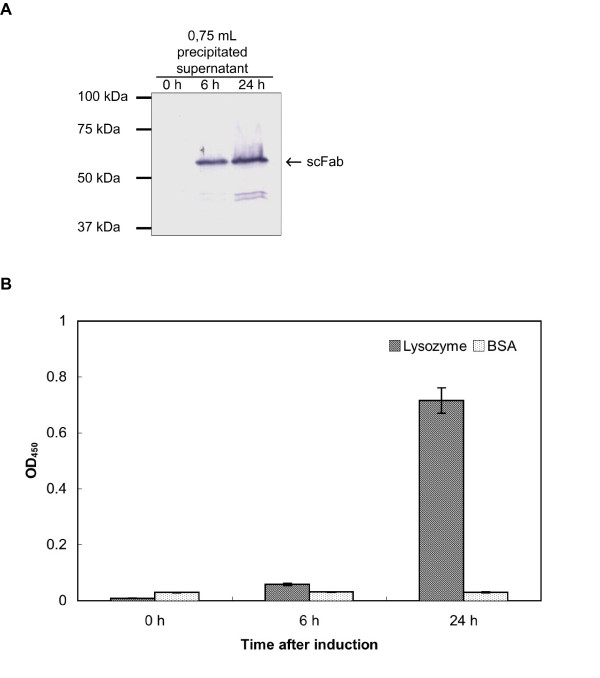
Analysis of functional anti-lysozyme D1.3 scFab after 0, 6 and 24 h of production. **A **Ammonium sulfate precipated scFabs from 0.75 mL supernatant were separated by reducing SDS-PAGE (10%) and detected using mAb mouse anti-His and goat anti-mouse IgG AP (Fc specific). **B **Antigen binding ELISA with 50 μL culture supernatant from a production in 100 mL scale. Mean values and standard deviations of data obtained from three different experiments are given. Antigens: 1 μg/well lysozyme or 1 μg/well control protein BSA. The D1.3 scFabs were detected using mAb mouse anti-His and goat anti-mouse IgG HRP (Fc specific).

The D1.3 scFab was produced in *B. megaterium *in shaking flask for 24 h after induction with xylose. Samples were taken from the supernatant after 0 h, 6 h and 24 h after induction were ammonium sulfate precipitated and aliquots were analysed by SDS-PAGE and immunoblot (Fig. 2A) and ELISA on lysozyme (Fig. 2B). After 6 hours of production first scFab were detected by immunostaining, whereas almost no specific activity was determined by ELISA. Best production and function was observed after 24 hrs.

**Figure 3:**
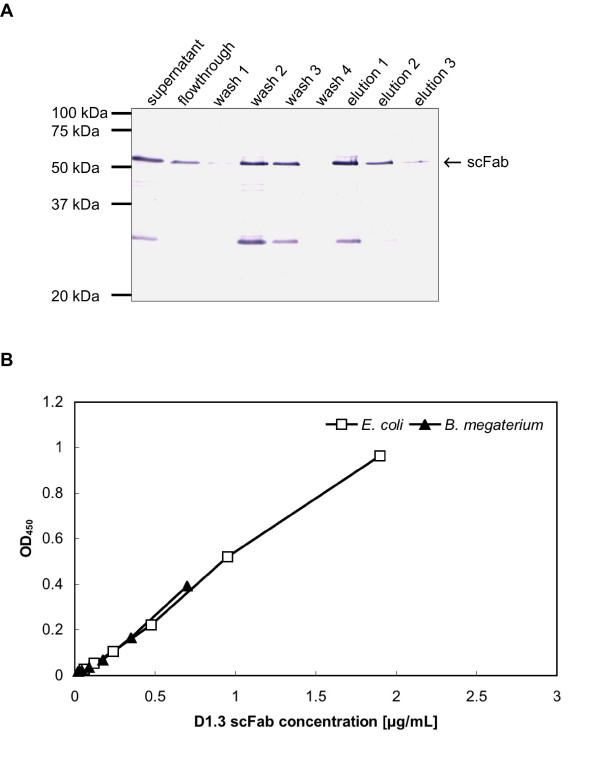
Purification and analysis of anti-lysozyme D1.3 scFabs. **A **Westernblot and immunostain of the combined supernatan of 6 independent productions, wash fractions and elution fractions of the IMAC purification. Samples were separated by reducing SDS-PAGE (12%) and detected using mAb mouse anti-His and goat anti-mouse IgG AP (Fc specific). **B **Antigen binding ELISA of purified scFab produced in *B. megaterium *or *E. coli*, performed as described in figure 2.

For purification, the supernatant of the D1.3 scFab production in *B. megaterium *was precipitated using ammonium sulfate and the resolved pellet was purified by immobilized metal affinity chromatography (IMAC) (Fig. 3A). For comparision, the D1.3 scFab produced in *E. coli *was isolated from both the periplasmic space and the medium supernatant. In total, 3,5 μg/L scFab D1.3 were isolated from *B. megaterium *and 9,5 μg/L scFab were isolated from *E. coli*. The specific activity of the D1.3 scFab produced in both production hosts were compared by ELISA on antigen (Fig. 3B). The specific activity determined by ELISA of the purified scFab was similiar for both production systems.

### Production and analysis of the anti-CRP scFv in *Bacillus megaterium*

**Figure 4:**
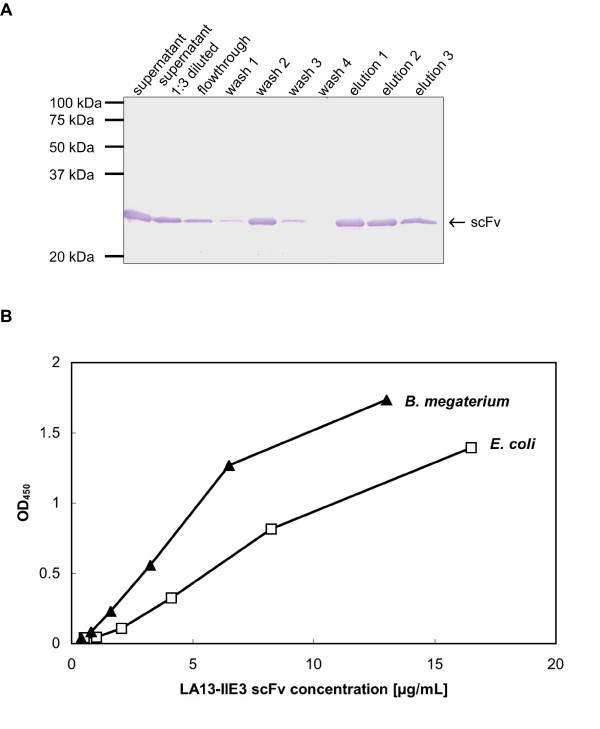
Purification and analysis of anti-CRP LA13-IIE3 scFvs. A Westernblot and immunostain of the combined supernatant of 3 independent productions, wash fractions and elution fractions of the IMAC purification. Samples were separated by reducing SDS-PAGE (12%) and detected as described in figure 3. B Antigen binding ELISA of purified anti-CRP scFv produced in *B. megaterium *or *E. coli*. Antigens: 100 ng/well CRP or 100 ng/well control protein BSA, detection as described in figure 2.

To verify the results of the D1.3 scFv production in *B. megaterium *[18], an anti-CRP scFv was produced in *B. megaterium*. The scFv was purified from the precipitated supernatant using IMAC (Fig. 4A). In parallel, the anti-CRP scFv was produced in *E. coli*. In total, 390 μg/L anti-CRP scFv were isolated from *Bacillus megaterium *and 550 μg/L anti-CRP scFv were isolated from *E. coli*. The specific activity of the LA13-IIE3 scFv produced in both production hosts were compared by ELISA on antigen (Fig. 4B). The specific activity of the scFv was higher when produced in *B. megaterium*.

## Discussion

The Gram-negative bacterium *E. coli *is the best characterized bacterial production host and is a well established production host for recombinant antibody fragments. The yield strongly depends on the individual antibody and the production system, e.g. shake flask or fermenter, e.g. 300 μg/L were reported for an anti scorpion toxin scFv in shake flask [19], but up to 2.5 g Fab fragments/L were obtained from high cell fermentation [20]. In *E. coli*, the correct formation of disulphide bonds, a prerequisite for functional antibodies and antibody fragments, is only possible in the oxidizing environment of the perisplamic space. Therefore, the produced antibody fragments have to be isolated from the periplasm, so usually only a fraction accumulates in the medium supernatant. But in many cases antibody fragments can be isolated from the medium supernatant [21-23]. The advantage of Gram-positive bacteria is the lack of the outer membrane. Hence, all produced antibody fragments can directly be harvested from the medium supernatant. Gram-positive bacteria already have been succesfully used for the production of antibody fragments, like *B. subtilis *[15,16], *B. brevis *[13,14] and very recently, *B. megaterium *[18] was shown to be able to secrete functional scFv fragments.

Here, a new antibody format, the scFab, was produced in *B. megaterium *to analyse the capability of this production system to assemble more complex antibody fragments with a higher molecular weight. Functional anti-lysozyme D1.3 scFabs were successfully produced using *B. megaterium*. The total amount of the scFab was significantly lower compared to the production of the corresponding scFv fragment [18]. This was expected due to the double molecular size and double amount of disulphide bonds of the scFab. Additionally, the scFab was produced in *E. coli *to compare the yield and the specific activity. The total yield of the production in *B. megaterium *is lower than in *E. coli*. Whereas, the specific activity of the purified scFab produced in *B. megaterium *is comparable to *E. coli*. *B. megaterium *is next to *E. coli *and *P. pastoris *the third production host which was shown to be suitable for the production of scFabs.

Consequently, the production of another scFv, an anti-CRP scFv LA13-IIE3, was analysed. The scFv LA13-IIE3 was produced in similar amounts compared to D13 scFv in *B. megaterium*. The total amount of purified LA13-IIE3 was slightly higher in *E. coli*. However, the specific activity of the scFv produced in *B. megaterium *was higher when compared to the *E. coli *production. A similiar difference was observed before for the anti-lysozyme antibody D1.3 [18].

*E. coli *production systems were optimized for the production of heterologous proteins for more than two decades [24] and used for the expression of recombinant antibodies since 1988 [4,5,25]. The new production host *B. megaterium *has to be optimized for the production of antibodies to increase the yield. The highest production yields of *E. coli *were reached using high cell fermentation, e.g. Chen et al. [20]. Fermentation of *B. megaterium *for the production of non heterologous proteins was shown, e.g. Sindhu et al. [26]. Because of the higher stability of plasmids in *B. megaterium *compared to *B. subtilis *during growth, *B. megaterium *may well be better suited for the production of recombinant proteins in fermenters [17].

In conclusion, the production host *B. megaterium *offers an alternative to *E. coli *for the production of recombinant antibody fragments which has to be further evaluated.

## Conclusion

*B. megaterium *is able to produce more complex antibody formats like the novel scFab. It shows less total productivity but similiar product quality compared to *E. coli*. In comparision to *E. coli*, *B. megaterium *production is still not very well examined and need further optimisation.

## Methods

### Transformation of *B. megaterium*

Transformations of non-sporulating *B. megaterium *strain MS941 [27] were performed as described by Barg *et al.*[28].

### Production and export of scFab and scFv using *B. megaterium*

The anti-lysozyme scFab D1.3 and anti-CRP scFv LA13-IIE3 were produced in 500 mL shaking flasks. 3 × 100 mL TB medium + 10 μg/mL tetracycline [29] for scFv LA13-IIE3 or 6 × 100 mL for scFab D1.3 were inoculated with 1 mL overnight culture at 37°C and 250 rpm. The induction was started by adding 0.5% xylose at O.D._600 nm _= 0.3 – 0.4. The cultures were grown at 250 rpm and 41° for 24. The supernatant was directly used for ELISA. For SDS-PAGE analysis and protein purification the proteins of the supernatant were precipitated using 440 g/L ammonium sulfate.

### Production of scFab and scFv in *E. coli*

The anti-lysozyme scFab D1.3 and anti-CRP scFv LA13-IIE3 were produced in 1 L shaking flasks using the vector pOPE101 [30] and the *E. coli *strain XL1-Blue MRF' (Stratagene, Amsterdam, Netherland). Briefly, 300 mL 2 × TY + 100 mM glucose + 100 μg/mL ampicillin were inoculated with an overnight culture yield to O.D._600 nm _= 0.1 and cultured at 37°C and 250 rpm. The scFv and scFab production was induced by adjusting to 50 μM isopropyl-beta-D-thiogalacto-pyranoside (IPTG) at O.D._600 nm _= 0.5 and shaking for 3 h at 30°C. Bacteria were harvested by 15 min at 4225 × g and 4°C. The supernatant was used for ammonium sulfate precipitation (see below). Bacteria pellets were resuspended in 1/10 culture volume ice cold PE buffer, pH 8 (20% sucrose, 50 mM Tris, 1 mM EDTA) and incubated for 20 min on ice, interrupted by short vortexing every 2 min. Subsequently the bacteria were pelleted for 30 min at 30,000 × g and 4°C. The supernatant (periplasmic fraction) was stored at -20°C. The remaining supernatant from the first bacteria centrifugation was precipated using 440 g/L ammonium sulfate, stirred 1 h at 4°C and centrifuged for 30 min at 13689 × g and 4°C. The protein pellet was dissolved in 35 mL/L culture phosphate buffered saline (PBS) [29]. The periplasmic fraction and the precipitated supernatant were combined and dialysed over night against PBS at 4°C.

### Immobilized metal affinity chromatography (IMAC) purification of antibody fragments

Antibody fragments were purified from *E. coli *or *B. megaterium *derived material by affinity chromatography using IMAC. Chromatography using 0.5 mL Chelating Sepharose Fast Flow (GE Healthcare, Munich, Germany) was performed according to the manufacturers' instruction. The protein solution was adjusted to 10 mM imidazol containing buffer pH7.4 (20 mM Na_2_HPO_4_, 500 mM NaCl, 10 mM imidazol) for loading. The column was washed once with 10 mM imidazol, once with 20 mM for scFv purification or with 50 mM for scFab purification, then once with 50 mM imidazol buffer and once with PBS. The elution was done in three steps, twice elution with 20 mM Na_2_HPO_4_, 500 mM NaCl and 250 mM imidazol and finally PBS with 100 mM EDTA were used for the elution.

### Antigen binding ELISA

Maxisorb MTPs (Nunc, Wiesbaden, Germany) were coated with either 1 μg hen egg white lysozyme, 100 ng CRP (BiosPacific, Emeryville, California) or BSA as control protein in 100 μL PBS per well overnight at 4°C. Coated wells were washed three times with PBST (PBS + 0,1% (v/v) Tween 20) and blocked with 2% (w/v) skim milk powder (Roth, Karlsruhe, Germany) in PBST for 1.5 h at RT, followed by three times washing with PBST. Soluble antibody fragments were diluted in 100 μL blocking solution and incubated for 1.5 h, followed by three times washing with PBST. Soluble antibody fragments were detected with mAb mouse anti-penta His-tag (1:10000) (Qiagen, Hilden, Germany) and polyclonal goat anti-mouse IgG (Fc specific) conjugated with horseradish peroxidase (HRP) (1:10000) (Sigma, Taufkirchen, Germany) and visualised with 100 μL TMB (3,3',5,5'-tetramethylbenzidine) substrate. The staining reaction was stopped by adding 100 μL 1 N sulphuric acid. The absorbance at 450 nm subtracted by the absorbance of scattered light at 620 nm were measured using a microtitre plate reader SUNRISE (Tecan, Crailsheim, Germany).

### SDS-PAGE and Immunoblot

Soluble antibody fragments were separated by SDS-PAGE [31] and blotted onto polyvinylidene fluoride (PVDF) membrane (Millipore, Schwalbach, Germany). The membrane was blocked with 3% (w/v) skim milk powder in PBS for 30 min at RT. For the detection of soluble antibody fragments the mAb mouse anti-penta His-tag (1:2000) was used as first antibody and goat anti-mouse IgG (Fc specific) conjugated with alkaline phosphatase (AP) (Sigma, Taufkirchen, Germany) (1:5000) was used as second antibody. The blot was visualised using NBT/BCIP substrate reaction.

## Authors' contributions

EJ performed the experiments and helped to draft the manuscript. LA generated the anti-CRP antibody. MH drafted the manuscript and participated in the design and coordination of the study. TS and SD participated in the design and coordination of the study and helped to draft the manuscript. SD conceived the project and wrote the grant application. All authors read and approved the final manuscript.
